# Correspondence

**Published:** 1909-02

**Authors:** 


### Correspondence.

### The Sectarian Medical College.

*Editor Texas Medical Journal:*

"Where one has to do with an art the end of which is the saving of human life, any neglect to make one's self thoroughly master of it is a crime."

There has been much controversy over the amalgamation of the different schools and sects of medicine. Legislation, forced methods and honeyed words have failed to accomplish this much-desired condition of matters medical. Every effort has resulted in drawing the lines of demarkation more firmly. Where there is a principle involved, a surrender is impossible to an honest man.

The homeopaths not only believe but know that they have a therapeutic law that applies as well today as when first promulgated and one that will continue indestructible for all time. To apply this law one must not only be familiar with it but must be conversant with the action of remedies. To master both there must be colleges for teaching their distinctive methods.

In the November issue of the *Journal of the American Medical Association* is an article by Arthur C. Jacobson, M. D., advising that all methods taught in the "irregular schools" be taught in all medical colleges. The writer excepts homeopathy, seemingly prefering the method adopted by the University of Michigan and several other of the Northern States, viz., chairs of homeopathy added to the curriculum of the State university medical college, thereby affording all students desiring to study homeopathy an opportunity to do so and at the same time avail themselves of the other branches taught in the college. This obviates the necessity for attendance on an independent homeopathic college.

With every State university medical college teaching the homeopathic branches, the independent schools would gradually draw out, and one can readily imagine a better feeling existing between the graduates of these State schools than between the graduates of the independent schools. Until all doctors are conversant with homeopathic therapeutics there will continue to be a separate and distinct school of medicine practicing this system. Physicians of the State can avail themselves of post-graduate courses in these schools without compromising their dignity, as in case of attendance upon independent homeopathic colleges.

With the free State medical schools teaching both branches of therapeutics, each State would educate most of its students at home, helping to build up the State schools, bringing about the elimination of all sectarian schools, so much desired by Dr. Jacobson.

As time goes on our State universities will far outstrip the independent colleges and all medical colleges must succumb except the sectarian colleges; these will continue at the same old stand unless their distinctive branches are taught equally as well in the State medical colleges. When this shall be accomplished they, too, will find it difficult to exist.

Richard Lloyd Jones in the September 12th issue of *Collier's* has furnished us an admirable article on "The Commonwealth Colleges." He compares the commonwealth college with the independent or endowed college. Six States only are without State universities, while many universities have the medical branches. The writer classes "Harvard, Yale and Princeton as types of the endowed American university." "The universities of Illinois,

Nebraska and Texas are types of the commonwealth institutions." "Harvard is over two and one-half centuries old, Yale is more than two centuries old and Princeton has passed her one hundred and fiftieth birthday; the universities of Illinois and Nebraska are neither forty years old and the University of Texas is but twenty-five. Twenty years ago the total enrollment of Yale, Harvard and Princeton was 3593; the total enrollment of Illinois, Nebraska and Texas was 1156. Today, Yale, Harvard and Princeton have a student population of 9172; while Illinois, Nebraska and Texas number 9925 students"—a net gain in favor of the commonwealth colleges of 2890. When we consider that the endowed colleges are not increasing their enrollment now and the commonwealth colleges were never increasing so rapidly, it is reasonable to expect a much greater gain during the next ten years in favor of the commonwealth college than there has been during the past twenty years. During the past five years Harvard has found her registration diminishing, caused, according to President Eliot, by the growth of the State universities. Twenty-five years hence will see but a limited number of endowed colleges in existence. Harvard, Yale, Princeton, Cornell or Columbia and others will have become State schools. There is now a move on foot to effect this. The medical colleges will follow as the free State medical schools will keep pace with the other branches of the State universities. This will leave only the sectarian medical colleges to be reckoned with. When the homeopathic medical students can obtain as good an education in the free State schools as in their endowed schools, there will be no need for the latter.

W. D. Gorton, M. D.

Austin, Texas, February 1, 1909.

---

### Hospital and Clinical Notes.

*Editor Texas Medical Journal:*

My hospital and clinical advantages have been very gratifying in the North, and a few notes of the same may be of interest to the many medical readers of the "Red Back." During my absence from home I have visited Racine, Wis.; Chicago, Indianapolis, South Bend and Terre Haute, Ind. I did not visit hospitals or clinics at South Bend, my chief object of interest there being the one-quarter million dollar Y. M. C. A. building presented to the organization of South Bend by Studebakers. Any physician or hygienist, whether interested in the moral, religious and intellectual work of the Y. M. C. A. or not can not fail to be pleased with the hygienic and social features of what I suppose is the completest building of its kind in the world. It is worth a trip to South Bend to see.

At this writing I can give you only a hasty view of hospital and clinical affairs at Terre Haute, Ind., and my advantages and observations which have been very ample, interesting and advantageous, as every courtesy has been shown me.

A young ladies' seminary was converted into St. Anthony's hospital, to which large additions have been made from time to time, the largest and most important of which is now being rapidly completed, which will increase the capacity of the institution to nearly two hundred beds. The operating amphitheater, on the third and topmost floor, when completed, will be the most perfect and up-to-date arrangement of the kind in any institution I have visited, in this country, or in France.

This city and extensive surrounding farming and mining country furnish a wide field for surgery; and skillful and enterprising men are not slow to seize opportunities and meet the demands.

The following statement will give some idea of what has been done during the past two years at St. Anthony's:

Dr. L. J. Willien who is really the organizer of the hospital, and the oldest physician connected with it, reports the following surgery in 1907:

Appendectomies, 24; laparotomies for removal of tubes and ovaries, 65; herniotomies, 7; hysterectomies, 6; gall-stones, 7; gunshot in abdomen, 1; septic kidney, 1; tubercular ascites, 1; tubal pregnancy, 1. Total number of operations in hospital during 1907, 511. Total number of abdominal operations, 140. Number done by Dr. L. J. Willien, 113.

During the year 1908, from January 1st to December 1st, there were done, as follows: Appendectomies, 44; laparotomies, resection of bowel, 2; removal of tubes and ovaries, 55; herniotomies, 3; hysterectomies, 3; gall-stones, 7; intestinal obstruction, 3; peritonitis, 1; stone in bladder, 2. Total number of operations from January 1, to December 1, 1908, 594. Number operated on by Dr. L. J. Willien, 120.

On the morning of December 11, 1908, I witnessed a very interesting abdominal section on a colored woman, 38 years of age, the mother of two children, the youngest being 17 months old. Her father lived to be 100 years old. Otherwise family history was negative. She had been ailing one year. The development of abdominal tumor was slow. It was movable to a certain extent, and extended over the entire abdominal region. Its feel was irregular and nodular with considerable fluctuation.

On making an incision of about four inches in length, a cancerous condition of the lesser omentum was disclosed, followed by a gush of blood of about a quart, which had filled the cavity, and proceeded from a mesenteric vein eaten off by the cancer. Of course, nothing could be done but to close up the cavity as quickly as possible, it being evident that she could survive but a short time.

I have had especial advantages in the line of dermatology, of all of which I have taken note; but none of the cases I have seen were quite equal to some I have treated at home.

J. W. CARHART,
Austin, Texas.

